# Construction and validation of educational materials for the prevention of metabolic syndrome in adolescents^1^

**DOI:** 10.1590/1518-8345.2024.2934

**Published:** 2017-10-05

**Authors:** Ionara Holanda de Moura, Antônia Fabiana Rodrigues da Silva, Aparecida do Espírito Santo de Holanda Rocha, Luisa Helena de Oliveira Lima, Thereza Maria Magalhães Moreira, Ana Roberta Vilarouca da Silva

**Affiliations:** 2 MSc, Professor, Universidade Federal do Piauí, Picos, PI, Brazil.; 3 Undergraduate student in Nursing, Universidade Federal do Piauí, Picos, PI, Brazil.; 4 Undergraduate student in Nutrition, Universidade Federal do Piauí, Picos, PI, Brazil.; 5 PhD, Adjunct Professor, Universidade Federal do Piauí, Picos, PI, Brazil.; 6 PhD, Adjunct Professor, Universidade Estadual do Ceará, Fortaleza, CE, Brazil.

**Keywords:** Validation Studies, Educational Technology, Metabolic Syndrome X, Health Education, Adolescent

## Abstract

**Objective:**

To develop and validate an educational technology focused on prevention of metabolic syndrome among adolescents.

**Methods:**

This was methodological research. Using an integrative review, the available publications on the subject were analyzed. Then, this knowledge was used to describe the theoretical content and, with the help of a graphic designer, the art and layout of the pages were developed. In the third phase, the booklet was evaluated and validated by 21 specialists and 39 adolescents. Data collection included three different questionnaires, according to the focus of evaluation of each group of participants, analyzed for reliability (Cronbach’s Alpha) and agreement by Infraclass Correlation Coefficient.

**Results:**

The mean score attributed by technical content experts was 91.7%, and the content validity index, measured by experts responses, was 0.98, showing high reliability and agreement. In addition, the level of agreement of the positive responses given by adolescents was 88.4%.

**Conclusion:**

the educational booklet has proved to be a valid and reliable tool to be used for promoting adolescent health.

## Introduction

The worldwide population, including within Brazil, underwent a process of socioeconomic transformation in the last century. The advent of modernization, brought by the innumerable (re)developed technologies, favored the improvement of quality of life and greater convenience in daily life; however, it also affected eating habits and energy expenditure, which crucially influences the health-disease process.

In addition to the issue of genetic predisposition, individuals are at risk of developing obesity, diabetes mellitus (DM), systemic arterial hypertension (SAH), and metabolic syndrome (MS)^1^. The MS represents the most common metabolic abnormality, currently, and is responsible for the majority of cardiovascular events within the population^2^. 

Due to its magnitude, multiple population studies have been developed worldwide on MS, demonstrating a prevalence of: 24.3% in European and American adults^3^; 13.7% in children of both sexes in Cutitiba, (PR), Brazil^4^; an overall rate of 32.5% (95% CI: 30.1% - 35.0%), with no significant difference between sexes was identified in a systematic review^5^. 

These data revealed that MS exists in a considerable portion of the population, including among the youngest individuals, and that it is fundamental to implement public health programs focused on these aspects. In this context, for effectiveness of primary prevention, health education is a fundamental tool for the development of self-care and coping with the health-disease process, through exchange of popular and scientific knowledge that reconstructs meanings and attitudes^6^.

Health education is frequently associated with the use of printed educational materials, while the concomitant application of verbal guidance along with what is written makes the method more effective, and facilitates the comprehension of the subject, which promotes improvement in adaptation to the social/cultural context in which it is integrated^7^.

Considering these issues, this study aims to develop and validate an educational booklet aimed at prevention of metabolic syndrome among adolescents. Thus, it is expected to contribute to the knowledge of the population on the identification of risk factors and health promotion, as well as to guide professionals in developing health education actions.

## Methods

This was a methodological research study, conducted from March of 2015 to September of 2016. Specific guidelines were used for development and validation of guidance materials for health care^8^. 

In the first phase of the study (integrative review), the main publications on the prevention of metabolic syndrome in adolescents were analyzed, to compose the theoretical knowledge to be approached. Then, in the second phase, the art was developed using figures, formatting, configuration and layout of the pages by a graphic designer. 

The third phase (content validation) was performed by experts distributed in three distinct categories: eight content experts (researchers/professors in the area of MS, adolescent health, educational technologies and/or validation of instruments); six technical experts (professionals with experience in MS and adolescent health); and seven experts with professional design experience. To establish parameters for selection of participants, a system of expert classification was used, as well as snowball sampling, a technique widely used when the population has characteristics that are difficult to find.

The target audience, selected based on the results found in a previous study, was also accessed^9^. In the mentioned study, the author evaluated 421 students from the municipal public schools of the city of Picos-PI, regarding the risk factors for MS, finding 77 individuals with the presence of two or more of these factors. The individuals identified with increased risk, who met the inclusion criteria, were selected: ages between 14 and 17 years, regularly enrolled in one of the municipal public schools of the city in question, at least two risk factors for MS, and having availability for 20 to 30 minutes to participate by reading the booklet and responding to the evaluation questionnaire. The final sample consisted of 39 adolescents.

According to the specific focus of each group of participants, three instruments were used: the first focused on content and technical aspects, the second on design experts, and the third was geared toward the target audiences.

The assessment questionnaire sent to content and technical experts was the Suitability Assessment of Materials (SAM), which identifies the difficulty and convenience of educational materials. This is a three-point Likert scale in which a list of statements related to content, writing style, graphic illustration, presentation, motivation and cultural suitability are evaluated and rated as: one = inadequate, two = partially adequate, and three = adequate. The final scores should be equal to or greater than 60%, in order to consider the material adequate^10^. 

The assessment questionnaire for design experts presented questions regarding the characteristics of the illustrations. Similar to the previous questionnaire, the rating for the answers used a Likert scale type, in this case a four-point scale, defined as: one = totally disagree, two = disagree, three = agree, and four = totally agree. In this case, the Content Validity Index (CVI) was used to validate the instrument; value greater than 0.78 were accepted as valid^11^.

The last instrument, the assessment questionnaire for the target audience, contained items on organization, writing style, presentation and motivation domains; a minimum of 75% agreement on positive responses was required^12^.

The professional information about the experts, and the sociodemographic and clinical data of the adolescents, was organized in Excel 8.0 software; a descriptive analysis with calculation of absolute and relative frequencies was obtained, as well as measurement of central tendency (mean and median), and of dispersion (standard deviation and interquartile range).

The reliability of the instruments was analyzed using Cronbach’s Alpha and the Infraclass Correlation Coefficient (ICC) to evaluate agreement among the experts, at a significance level of 5%. Both statistical tests are presented in a ranged scale between 0 and 1, and values higher than 0.8 were acceptable^13^. These data were calculated using the Statistical Package for the Social Sciences (SPSS), version 20.0. After evaluation of all the suggestions made by experts and adolescents, the booklet was adapted according the needs and expectations of the population.

The research project was evaluated by the Research Ethics Committee of UFPI, through the Brazil Platform, obtaining approval in January of 2016, under protocol No. 1,394,242. The study was conducted in accordance with the ethical precepts established in Resolution 466/2012 of the National Health Council^14^. 

## Results

In the booklet development, the theoretical content was based on the knowledge available in the literature, ensuring complete information for adolescents, without being tiresome. This is reflected, for example, in the number of pages of the booklet, as well as in the choice of colors, which had the intention of being pleasant and attracting the reader’s attention.

Thus, instead of simply describing the information, it was decided to write a story, where the main character is a teenager who was diagnosed at risk for metabolic syndrome during a clinical assessment performed by health professionals at his school. At first, the character talks about his routine and how this condition was discovered. Then, by means of a conversation with the reader, basic concepts of MS are presented as well as methods for prevention; finally, he highlights the changes acquired in his family and school life.

To guarantee interactivity of the booklet for adolescents, two games throughout were inserted the history: a word-hunter to remember/memorize the components of MS, and a seven errors game to help with how to choose healthy foods. The booklet was develop for a young population, specifically adolescents; so all content choices and illustrations were defined according to the needs of these individuals.

Next, eight content experts were part of the booklet validation portion of the study; they were predominantly female (62.5%), with a mean age of 33.5+4.0 years. All the participants were nurses, and 75% pursued a doctoral-level degree. In addition, there were six technical experts, with a median age of 35.0 years, the majority of whom were female (66.7%); there was an equal participation of nurses, physicians and nutritionists.

Both content and technical experts responded to the SAM questionnaire. Table 1 shows each question, according to the attributes to be checked, and the number of individuals who rated the item as “inadequate”, “partially adequate”, and “adequate”.

**Table 1:** Content and technical expert evaluation regarding content, language, graphic illustrations, presentation, stimulation/motivation, and cultural adequacy of the booklet. Picos, Piauí, Brazil, 2016.

	**Inadequate**	**Partially adequate**	**Adequate**
**1 Content**			
**1.1 The objective is clear, facilitating immediate comprehension of the material**	**-**	**02**	**12**
**1.2 The content addresses behavioral information that helps to prevent MS**	**-**	**02**	**12**
**1.3 The material proposal is limited to the objectives**	**01**	**02**	**11**
**2 Language**			
**2.1 The reading level is appropriate for the reader’s comprehension**	**-**	**04**	**10**
**2.2 The style of conversation facilitates the understanding of the text**	**-**	**01**	**13**
**2.3 The information is clearly transmitted**	**-**	**01**	**13**
**2.4 The vocabulary uses common words**	**-**	**04**	**10**
**2.5 The learning is facilitated by topics**	**-**	**-**	**14**
**3 Graphic Illustrations**			
**3.1 The cover captures the reader’s attention and express the purpose of the material**	**-**	**02**	**12**
**3.2 The illustrations present fundamental visual messages so that the reader can understand the main outlines by himself**	**-**	**04**	**10**
**3.3 The illustrations are relevant**	**-**	**02**	**12**
**4 Presentation**			
**4.1 The organization of the material is adequate**	**-**	**04**	**10**
**4.2 The size and type of font promote an enjoyable reading experience**	**-**	**02**	**12**
**5 Stimulation/motivation**			
**5.1 Interaction occurs between text and/or figures and the reader. Facilitates problem solving, making choices, and/or demonstrating skills**	**-**	**03**	**11**
**5.2 The desired behavior patterns are modeled or well demonstrated**	**-**	**01**	**13**
**5.3 There is a motivation for self-efficacy**	**-**	**03**	**11**
**6 Cultural Adjustment**			
**6.1 The material is culturally appropriate to the logic, language and experience of the public**	**-**	**03**	**11**
**6.2 Displays culturally appropriate images and examples**	**-**	**-**	**14**

The data shows that only one expert evaluated item 1.3 as “inadequate”. Considering that no suggestions were added, and that the others experts evaluated the item as “adequate” (11) or “partially adequate” (02), no change was made in the booklet.

The items 2.5 and 6.2 showed agreement by all the experts, demonstrating that learning is facilitated by the topics, and that the images and examples are culturally appropriate. In addition, the most frequent classification attributed to the rest of the items assessed by the experts was “adequate”, reflecting their suggestions that the material was suitable.

**Figure 1:**
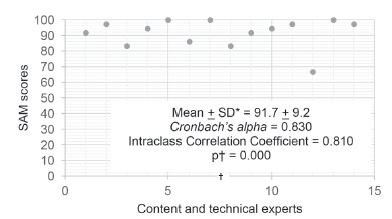
Percentage value of SAM score, referring to the evaluation of each content and technical expert. Picos, Piauí, Brazil, 2016.

Figure 1 shows the value score of the SAM, defined by the individual responses of each expert, calculated in percentages (line y - vertical), according to the respective participant (line x - horizontal).

*SD: standard deviation, †Significant p-value at the 5% level.

The mean score obtained was 91.7%, with a standard deviation of + 9.2%, with a minimum value of 66.7%, and a maximum value of 100.0%. This result was considered very satisfactory. Moreover, reliability (Cronbach’s alpha > 0.8) and agreement of responses (ICC > 0.8) was high, and statistically significant (p=0.000). Thus, the booklet was validated by content and technical experts.

The evaluation of the booklet was followed by the collaboration of seven professionals with design experience, mean age of 30.4 + 9.7 years, and male prevalence (71.6%). These individuals answered an adapted questionnaire, which enabled the qualification of the illustrations. Table 2 presents the answers attributed to each statement made, in which the experts could indicate, “Totally disagree”, “disagree”, “agree”, or “totally agree”, and the calculated CVI for each item.

**Table 2:** Design experts’ evaluation regarding the characterization of the graphic illustrations of the booklet. Picos, Piauí, Brazil, 2016.

	**Disagree**	**Agree**	**Totally agree**	CVI*
**1. They are appropriate for the target audience.**	**-**	**04**	**03**	**1,0**
**2. They are clear and easy to understand.**	**01**	**03**	**03**	**0,86**
**3. They are adequate in quantity and size.**	**-**	**02**	**05**	**1,0**
**4. They are related to the text and clarify the content.**	**-**	**04**	**03**	**1,0**
**5. The colors and shapes of the figures are appropriate.**	**-**	**04**	**03**	**1,0**
**6. They represent the daily life of adolescents.**	**-**	**04**	**03**	**1,0**
**7. The arrangement of figures is in harmony with the text.**	**-**	**04**	**03**	**1,0**
**8. The figures reveal the theme and are in a logical sequence.**	**-**	**03**	**04**	**1,0**
**9. They contribute to behavior and attitude change.**	**-**	**02**	**05**	**1,0**
**10. They are relevant to understanding the content.**	**-**	**02**	**05**	**1,0**

*CVI: Content validity index

The CVI calculated for each item reached the maximum value (1.0) for most of the cases; only item 2 obtained a different index (0.86), which did not compromise the results, as it was higher than the established limit. The global CVI computed was 0.98, the reliability (cronbach’s alpha = 0.917) and the concordance of the responses (ICC = 0.917) (p = 0.000) were classified as high. The booklet was also successfully validated by experts in the design area.

After adapting the material according to expert observations, the validation process was finalized with the contributions of the adolescents. It is worth emphasizing that this is not a clinical stage, but rather the verification with the population about the clarity, comprehension and relevance of the booklet content.

Adolescents aged 14 to 17 years participated in the study. The predominant sex was female (61.5%), and the majority self-reported as mixed ethnicity (61.5%). The family income of 61.5% was up to one times the monthly minimum wage; and 94.9% lived with their parents.

Adolescents were asked to respond to an adapted questionnaire^12^, seeking to analyze the organization, writing style, presentation and motivation of the booklet with the target audience. Table 3 shows the results obtained for each question, and the respective level of agreement for the answers.

**Table 3:** Target audience evaluation regarding the organization, writing style, presentation and motivation of the booklet. Picos, Piauí, Brazil, 2016.

	**Positive answers**		**Negative answers**		**Impartial answers**	
	**n**	**%**	**n**	**%**	**n**	**%**
**1. Organization**						
**1.1 Did the cover made you interested?**	**34**	**87.2**	**-**	**-**	**05**	**12.8**
**1.2 Is the content sequence adequate?**	**37**	**94.9**	**-**	**-**	**02**	**5.1**
**1.3 Is the structure of the educational booklet adequate?**	**35**	**89.7**	**-**	**-**	**04**	**10.3**
**2. Writing style**						
**2.1 Are the phrases (easy to understand/difficult to understand / do not know)?**	**39**	**100.0**	**-**	**-**	**-**	**-**
**2.2 The written content is (clear / confusing / do not know):**	**37**	**94.9**	**-**	**-**	**02**	**5.1**
**2.3 The text is (interesting / uninteresting / do not know):**	**38**	**97.4**	**-**	**-**	**01**	**2.6**
**3. Presentation**						
**3.1 The illustrations are (simple / complicated / do not know):**	**33**	**84.6**	**01**	**2.6**	**05**	**12.8**
**3.2 Do the illustrations complement the text?**	**32**	**82.1**	**-**	**-**	**07**	**17.9**
**3.3 Are the pages or sections organized?**	**35**	**89.7**	**-**	**-**	**04**	**10.3**
**4. Motivation**						
**4.1 In your opinion, will any teenager who reads this booklet understand?**	**33**	**84.6**	**01**	**2.6**	**05**	**12.8**
**4.2 Did you feel motivated to read the entire booklet?**	**26**	**66.7**	**01**	**2.6**	**12**	**30.8**
**4.3 Does the educational material address the issues necessary for adolescents to adopt a healthier lifestyle?**	**37**	**94.9**	**-**	**-**	**02**	**5.1**
**4.4 Did the educational booklet encourage you to act on or think about Metabolic Syndrome prevention?**	**32**	**82.1**	**02**	**5.1**	**05**	**12.8**

Among the four domains evaluated, writing style was the one that received the most positive responses: all the adolescents found the sentences easy to understand, 94.9% stated that written content was clear, and 97.4% that it was interesting, revealing the adequacy of the booklet for the target audience. The level of agreement of the positive responses ranged from 66.7% to 100.0% among the items addressed, totaling 88.4% in a general way, a sufficient score for validation of the educational booklet by the population.

## Discussion

In this study, the validation process was conducted with the participation of several professionals, representing a very favorable aspect, as it was possible to obtain significant specialized knowledge within the theme addressed by the material. Therefore, as in other research, a multidisciplinary, complete educational material was developed^15^ ^-^ ^16^.

The use of technology based on the active participation of the multiprofessional team is fundamental to improving the quality of care. In addition, the contributions suggested by the population were also fundamental to the process, because they represent the target of these health actions. Therefore, there was a concern to include the adolescents in the analysis of the booklet, as was done in other studies in the area^17^ ^-^ ^18^.

In general, the answers of the experts were concordant, as can be seen in the presented results. From the answers obtained in the SAM, given by content and technical experts (Figure 1), the mean score obtained was 91.7%, with high reliability and agreement.

Corroborating with these data, other methodological studies of educational technology development also validated their materials with high statistical indexes: the development of material for patients submitted to orthognathic surgery was concluded with an internal consistency of 0.972 and ICC of 0.601^7^; the booklet for healthy eating during pregnancy was validated with a level of concordance between the experts ranging from 0.818 to 0.954 for the evaluated items^19^.

The validation by experts in the design area also achieved excellent indices, with a global CVI equal to 0.98, as well as high reliability (Cronbach’s alpha=0.917) and high agreement for the answers (ICC = 0.917) (p = 0.000).

With a slightly lower index, a recent research conducted in Belém (PA), which validated an educational technology on postpartum care, obtained a global CVI equal to 0.81^20^. Another methodological research study conducted in Fortaleza (CE), which validated booklets for birth attendants, obtained a global CVI of 0.94^21^.

It is necessary to emphasize that, although the booklet was well evaluated by the experts, suggestions and observation were added, in order to guarantee a quality piece of educational material for the population; these details enrich the final product and improve its applicability, by reformulating information, replacing terms and reviewing illustrations^17^.

Thus, after suggestion of an expert, current and reliable data regarding the practice of physical activity was researched. The change in the booklet followed the recommendation of the World Health Organization (WHO)^22^, which indicates that physical exercise for children and adolescents, aged 5 to 17 years, should be at least 60 minutes daily of moderate or vigorous activity.

Two other experts emphasized the need for the protagonist of the story to be an overweight character, noting that this is a visible risk factor that is closely associated with MS. Indeed, the scientific literature accurately states this relationship, and the image of the character was adapted^23^ ^-^ ^24^.

In addition, images that express social inclusion were added to the booklet, as proposed by an expert. This was a lack of attention of the author, because the Brazilian Constitution is clear in guaranteeing everyone the right to inclusion, with equality and respect for the dignity of the human person and his social function. In this context, the State is responsible for creating conditions so that everyone can effectively be included in the society, and the participation of each citizen is fundamental in the design of healthy living environments^25^. 

Finally, the responses of the target audience showed a level of agreement of positive responses equal to 88.4%. However, it is necessary to highlight the motivation aspect, in which the answers were the least satisfactory, because although most respondents felt motivated to read the entire booklet, an expressive number of adolescents did not or were only partially motivated. The questionnaire was administered during testing periods at school, which probably had some influence on the answers, although the researcher explained that there would be no impact on the participants’ school performance for participating in the research; however, anxiety and a desire to return to the classroom were noticeable. 

From this perspective, the difficulty faced by the author in dealing with such a demanding public is clear. The educational booklet is a working proposal to support health education, which must be able to teach and stimulate the reader’s willingness to take responsibility for himself; nevertheless, the integrated use of this resource with other active methodologies is fundamental.

## Conclusion

At the end of the study, it is possible to conclude that the proposed objective was achieved. The educational booklet entitled “Metabolic Syndrome: How can it be prevented?” was validated regarding content, language, and presentation with experts, and for writing style, presentation, and understanding with the target population.

We hope that this educational booklet will actually be used for health promotion in the population, especially adolescents, so that they reflect on their current lifestyle and adopt the recommendations indicated in the material. From this perspective, nurses and other health professionals, as subjects committed to public health, play a critical role, whose responsibility is to act with a focus on health education and disease prevention. 

Finally, the reflection presented here is also focused on the environment for young people. Schools, families, and the community must be prepared to facilitate the routine of healthy eating, physical activity, and weight and stress control, as well as to reduce alcohol consumption and tobacco use*.*

## References

[1] Barbalho SM, Kawakubo AM, Souza KGF, Traldi JG, Mendes CG, Nery FM (2013). Estudo da presença de síndrome metabólica e relação com o histórico
familiar em escolares. Semina: Ciências Biológicas e da Saúde.

[2] Bortoletto MSS, Souza RKT, Cabrera MAS, González AD (2016). Síndrome Metabólica, componentes e fatores associados em adultos de 40
anos ou mais de um município da Região Sul do Brasil. Cad Saúde Coletiva.

[3] Scuteri A, Laurent S, Cucca F, Cockcroft J, Cunha PG, Mañas LR (2015). Metabolic Syndrome across Europe: different clusters of risk
factors. European J Preventive Cardiol.

[4] Titski ACK, Moser DC, Cieslak F, Mascarenhas LPG, Silva MJC, Leite N (2014). Frequência de Síndrome Metabólica em escolares. Pensar a Prática.

[5] Mitchell AJ, Vancampfort D, Sweers K, Van WR, Yu W, De Hert M (2013). Prevalence of Metabolic Syndrome and Metabolic Abnormalities in
Schizophrenia and Related Disorders - A Systematic Review and
Meta-Analysis. Schizopherenia Bull.

[6] Coelho MMF, Torres RAM, Miranda KCL, Cabral RL, Almeida LKG, Queiroz MVO (2012). Educação em saúde com adolescentes: compartilhando vivências e
reflexões. Cienc Cuid Saúde.

[7] Sousa CS, Turrini RNT (2012). Validação de constructo de tecnologia educativa para pacientes
mediante aplicação da técnica Delphi. Acta Paul Enferm.

[8] Echer IC (2005). The development of handbooks of health care guidelines. Rev. Latino-Am. Enfermagem.

[9] Carvalho RBN, Nobre RS, Guimarães MR, Teixeira SEXM, Silva ARV (2016). Risk factors associated with the development of metabolic syndrome in
children and adolescents. Acta Paul Enferm.

[10] Doak CC, Doak LG, Root JH (1996). Teaching patients with low literacy skills.

[11] Borges JWP, Moreira TMM, Rodrigues MTP, Souza ACC, Silva DB (2013). Content validation of the dimensions constituing non-adherence to
treatment of arterial hypertension. Rev Esc Enferm USP.

[12] Bispo GLR, Pedrosa EN, Wanderley RMM, Corrêa SMS (2012). Development and validation of the nursing instrument to postpartum
consultation. J Nurs UFPE.

[13] Fiel A (2009). Descobrindo a estatística usando o SPSS.

[14] Ministério da Saúde (BR) (2012). Resolução N° 466, de 12 de dezembro de 2012.

[15] Rabeh SAN, Gonçalves MBB, Cliri MHL, Nogueira PC, Miyazaki MY (2012). Construção e Validação de um módulo educativo virtual para terapia
tópica em feridas crônicas. Ver Enferm UERJ.

[16] Martins MC, Ferreira AMV, Nascimento LA, Aires JS, Almeida PC, Ximenes LB (2015). Influência de estratégia educativa na promoção do uso de alimentos
regionais. Rev RENE.

[17] Costa PB, Chagas ACMA, Joventino ES, Dod RCMT, Oriá MOB, Ximenes LB (2013). Construção e validação de um manual educativo para a promoção do
aleitamento materno. Rev. RENE.

[18] Guimarães FJ, Carvalho ARLF, Pagliuca LMF (2015). Elaboração e validação de instrumento de avaliação de tecnologia
assistiva. Rev Eletr Enferm.

[19] Oliveira SC, Lopes MVO, Fernandes AFC (2014). Development and validation of an educational booklet for healthy
eating during pregnancy. Rev. Latino-Am. Enfermagem.

[20] Teixeira E, Martins TDR, Miranda PO, Cabral BG, Silva BAC, Rodrigues LSS (2016). Educational technology on potpartum care: development and
validation. Rev Baiana Enferm.

[21] Teles LMR, Oliveira AS, Campos FC, Lima TM, Costa CC, Gomes FS (2014). Development and validating na educational booklet for childbirth
companions. Rev Esc Enferm USP.

[22] World Health Organization (2010). Global recommendations on physical activity for health.

[23] Faria FR, Faria ER, Faria FR, Paula HAA, Franceschini SCC, Priore SE (2014). Associação entre os componentes da síndrome metabólica e indicadores
antropométricos e de composição corporal em adolescentes. RevAssoc Bras Nutrição.

[24] Brito LMS, Galvanin CE, Amaral DC, Kato PVK, Cat MNL, Boguszewski MCS (2016). Influência da atividade físicas sobre critérios diagnósticos da
síndrome metabólica em estudantes. Arq Ciênc Saúde.

[25] Araújo LAD, Maia MA (2016). Cidade, dever constitucional de inclusão social e a
acessibilidade. Rev Direito Cidade.

